# Blood Platelets in the Progression of Alzheimer’s Disease

**DOI:** 10.1371/journal.pone.0090523

**Published:** 2014-02-28

**Authors:** Nina S. Gowert, Lili Donner, Madhumita Chatterjee, Yvonne S. Eisele, Seyda T. Towhid, Patrick Münzer, Britta Walker, Isabella Ogorek, Oliver Borst, Maria Grandoch, Martin Schaller, Jens W. Fischer, Meinrad Gawaz, Sascha Weggen, Florian Lang, Mathias Jucker, Margitta Elvers

**Affiliations:** 1 Department of Clinical and Experimental Hemostasis, Hemotherapy and Transfusion Medicine, Heinrich-Heine-University, Düsseldorf, Germany; 2 Medizinische Klinik III, Kardiologie und Kreislauferkrankungen, Eberhard-Karls-Universität, Tübingen, Germany; 3 Department of Cellular Neurology, Hertie-Institut for Clinical Brain Research, Eberhard-Karls University, Tübingen, Germany; 4 DZNE, German Center for Neurodegenerative Diseases, Tübingen, Germany; 5 Department of Physiology, Eberhard-Karls University, Tübingen, Germany; 6 Department of Neuropathology, Heinrich-Heine-University, Düsseldorf, Germany; 7 Institut für Pharmakologie u. Klinische Pharmakologie, Universitätsklinikum der Heinrich-Heine-Universität, Düsseldorf, Germany; 8 Department of Dermatology, Eberhard-Karls University, Tübingen, Germany; Massachusetts General Hospital/Harvard Medical School, United States of America

## Abstract

Alzheimer’s disease (AD) is characterized by neurotoxic amyloid-*ß* plaque formation in brain parenchyma and cerebral blood vessels known as cerebral amyloid angiopathy (CAA). Besides CAA, AD is strongly related to vascular diseases such as stroke and atherosclerosis. Cerebrovascular dysfunction occurs in AD patients leading to alterations in blood flow that might play an important role in AD pathology with neuronal loss and memory deficits. Platelets are the major players in hemostasis and thrombosis, but are also involved in neuroinflammatory diseases like AD. For many years, platelets were accepted as peripheral model to study the pathophysiology of AD because platelets display the enzymatic activities to generate amyloid-*ß (*A*ß)* peptides. In addition, platelets are considered to be a biomarker for early diagnosis of AD. Effects of *Aß* peptides on platelets and the impact of platelets in the progression of AD remained, however, ill-defined. The present study explored the cellular mechanisms triggered by A*ß* in platelets. Treatment of platelets with A*ß* led to platelet activation and enhanced generation of reactive oxygen species (ROS) and membrane scrambling, suggesting enhanced platelet apoptosis. More important, platelets modulate soluble A*ß* into fibrillar structures that were absorbed by apoptotic but not vital platelets. This together with enhanced platelet adhesion under flow *ex vivo* and *in vivo* and platelet accumulation at amyloid deposits of cerebral vessels of AD transgenic mice suggested that platelets are major contributors of CAA inducing platelet thrombus formation at vascular amyloid plaques leading to vessel occlusion critical for cerebrovascular events like stroke.

## Introduction

Deposits of A*ß* are the characteristic pathological hallmarks of Alzheimer’s disease (AD), an age-related neurodegenerative disorder and the most common form of senile dementia [1]. Recent studies provided strong evidence that misfolding and aggregation of A*ß* is a crucial event in the pathogenesis of AD [1], followed by neuron loss and declined cognitive and memory capability, affecting about 26 million people worldwide with tendency to rise [2]. In recent years, AD has been recognized to be a more intriguing disorder affecting other peripheral tissues beside the brain demonstrating that alterations in AD patients occur not only in the central nervous system but also in blood vessels and blood cells leading to amyloid-*ß* deposits in cerebral vessels known as cerebral amyloid angiopathy (CAA) [3]. CAA plays an important role in the severity of AD pathology because it induces degeneration or even destruction of the vessel wall and affects cerebral blood flow [3]. Besides CAA, recent studies provide strong evidence that AD is strongly related to the vascular system. Different clinical trials provide evidence that AD is related to vascular diseases such as stroke [4], atherosclerosis [2] and hypertension [5]. These vascular risk factors increase the risk for AD [6], and moreover, cerebrovascular dysfunction occurs in AD patients leading to alterations in blood flow that might play an important role in AD pathology with neuronal loss and memory deficits [7], [8]. To date the mechanisms how A*ß* could alter thrombosis and hemostasis is not known but it is hypothesized that A*ß* might be involved in blood flow and blood vessel function [9], [10].

Platelets are anuclear cells crucial for hemostasis at sites of vascular injury. However, uncontrolled platelet activation can lead to acute vessel occlusion leading to myocardial infarction and stroke [11]. In the last years it has become increasingly evident that platelets are also important players in inflammation, angiogenesis and tumor progression [11]. Furthermore, platelets express the amyloid precursor protein (APP) and display the complete enzymatic machinery to process APP proteins into amyloid-*ß* (A*ß*) peptides through a pathway known in the brain [2]. Thus platelets have been considered to be a good model to study neurodegenerative diseases like AD for decades.

In the last years, platelets were used to study metabolic mechanisms relevant in the central nervous system and related to AD. Signalling pathways in platelets leading to platelet activation and aggregation have been described to also modulate APP processing [12], [13]. Platelets contain high levels of APP with different expression pattern compared to the brain [14] but represent the fundamental repository of A*ß* in blood that might contribute to the accumulation of A*ß* in brain and its vasculature [15]–[17]. So far, the physiological role of platelet APP and its metabolites is, however, ill-defined. Different studies propose that APP may act as a receptor on the platelet surface [18] and might be involved in Ca^2+^ mobilization [19].

Platelets can release A*ß* and become activated by synthetic A*ß* peptides [20]. The processing of APP in platelets of AD patients is altered compared to control subjects suggesting since several years that platelets may be a biomarker for the diagnosis of AD. For instance, Rosenberg and co-workers showed altered APP processing in platelets of AD patients and found platelet APP isoform ratios to correlate with declining cognition in AD [21], [22].

Platelets are major players in vascular diseases associated with AD such as atherosclerosis and stroke [11]. In addition, blood flow alterations induced by CAA or AD-related vascular diseases with consecutively hypoperfusion induced by vessel occlusion point to a second effect of A*ß* in brain beside its neurotoxic effects [3]. This suggests that platelets not only mirror AD related events in the brain but might influence the progression of AD. Although much effort has been made to understand the role of platelets in AD, the molecular mechanisms involved and the impact of platelets in AD is not well understood.

The present study explored the mechanisms underlying the effects of amyloid-*ß* on platelets. It is shown that amyloid-*ß* exposure leads to platelet activation, A*ß* release, ROS generation and membrane scrambling. Furthermore, we provided strong evidence that platelets contribute to CAA by modulation of soluble into fibrillar A*ß* and facilitation of platelet adhesion and accumulation at vascular amyloid-*ß* deposits leading to full occlusion of affected cerebral vessels critical for ischemic cerebrovascular events like stroke.

## Results

### Contact of Platelets with Synthetic Aß Leads to Platelet Aß Production

**Figure 1:**
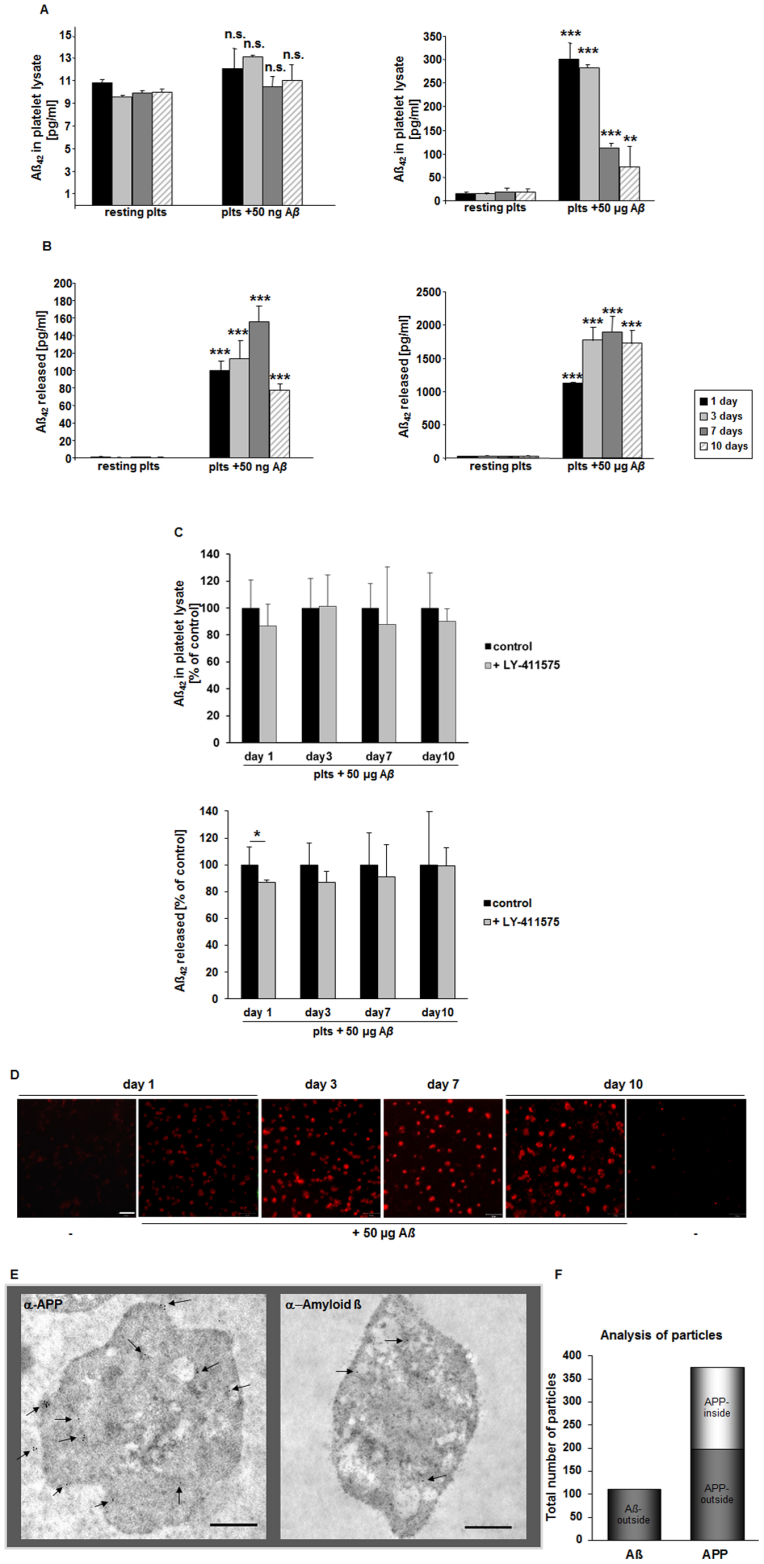
Contact of platelets with soluble A*ß* leads to A*ß* production in human platelet culture. **(**A) Accumulation of A*ß* (1–42) in activated human platelets was measured at indicated time points by sandwich ELISA assay. Bar graphs depict mean values ±SEM (n = 4) of Aß1–42 levels in resting and A*ß* (50 ng/ml, left panel, 50 µg/ml right panel) stimulated platelets. (B) A*ß* (1–42) levels measured in supernatants of resting and A*ß* stimulated platelets after 1, 3, 7 and 10 days in culture. **p<0.01 and ***p<0.001 indicates statistically significant difference to values in the absence of A*ß*, n.s. not significant. (C) A*ß* (1–42) was measured in platelet lysate and supernatant after pre-treatment with the γ-secretase inhibitor LY 411575. Bar graphs depict mean values ±SEM (n = 3), *p<0.05. (D) Platelets were isolated and stimulated with 50 µg/ml A*ß* for indicated time points. A*ß* was detected by confocal microscopy using an antibody directed to A*ß* (6E10, Covance). (E) Electron microscopy with postembedding immunogold labeling in human platelets at 24 h in the presence of A*ß*. Expression of APP at the plasma membrane and in granules was evident (see arrowheads, left panel). A*ß* was only marginally detected at the plasma membrane (see arrowheads, right panel). Scale bar 1 µm. (F) Statistical analysis of gold-labeled APP and A*ß*.

Recent studies provide evidence that platelets are able to process and release A*ß* peptides, processes enhanced by platelet stimulation with the platelet agonist thrombin [23]. To investigate A*ß* production of platelets in further detail, we isolated human and murine platelets, respectively and cultured platelets for 1–10 days with a synthetic soluble 40-residue A*ß* peptide to test whether platelets are affected by contact with soluble A*ß* found in blood by clearance of the CSF-brain system in normal individuals [24], [25]. Surprisingly, we found A*ß* (1–42) in platelet lysates after contact of platelets with soluble synthetic A*ß* that emerged to be concentration-dependent (Fig. 1A). While 50 ng/ml of soluble A*ß* does not influence the amount of A*ß* (1–42) in platelet cell lysates as measured by ELISA (10.8±1.77 pg/ml versus 12.05±0.28 pg/ml, day 1, Fig. 1A, left panel), incubation of platelets with high doses of A*ß* peptide (50 µg/ml) led to a strong increase of A*ß* (1–42) in platelet lysates (15.8±4.12 pg/ml versus 300.3±41.22 pg/ml, day 1; Fig. 1A, right panel). However, these results do not disclose if we measured A*ß* sticking to the platelet cell surface or if detected A*ß* is located to the cytoplasm or granules of platelets.

Consequently, we analyzed the release of A*ß* (1–42) from A*ß*-stimulated platelets and found a moderate increase in A*ß* levels when platelets were stimulated with low doses of A*ß* (50 ng/ml) (1.1±0.92 pg/ml versus 100.4±10.65 pg/ml, day 1; 0.4±0.21 pg/ml versus 112.9±21.33 pg/ml, day 3; 0.9±0.01 pg/ml versus 155.7±17.8 pg/ml, day 7; 0.1±0.02 pg/ml versus 77.8±7.11 pg/ml, day 10; Fig. 1B, left panel) while high doses of A*ß* peptide (50 µg/ml) induced a strong increase in A*ß* (1–42) release into the supernatant of platelets (24.1±6.02 pg/ml versus 1229.8±0.14 pg/ml, day1; 24.4±6.03 pg/ml versus 1939.9±200.81 pg/ml, day3; 26.9±2.0 pg/ml versus 2081.9±215.03 pg/ml, day7; 23.2±7.36 pg/ml versus 1892.5±195.44 pg/ml, day10; Fig. 1B, right panel). Thus, we conclude that contact of platelets with soluble synthetic A*ß* peptides is able to stimulate A*ß* (1–42) accumulation in/on platelets and also the release of A*ß* (1–42) from platelets in a time- and concentration-dependent manner. Control experiments (Fig. S1) showed that the A*ß* (1–42) ELISA we used is not entirely specific for Aß_42_. Therefore all results of Fig. 1A–B were corrected according to the respective controls (181.25±19.276 pg/ml in cultures with 50 µg/ml A*ß*; 1.345±0.076 pg/ml in cultures with 50 ng/ml A*ß*) to consider crossreactivity of Aß_40_ and Aß_42_.

In further experiments we treated platelets with the γ-secretase inhibitor LY 411575. Surprisingly, we found only less differences when we compared platelets that were treated with LY 411575 and untreated platelets (Fig. 1C) suggesting that most of the A*ß*_42_ measured by ELISA was not produced by platelets but is a result from platelet-mediated modification of A*ß*_40_ that was added to the platelet cell culture.

In addition, we activated platelets with A*ß* peptide and stained the cells using an A*ß* antibody (6E10) and found platelets to be positive for A*ß* within the whole culture period of 1 to 10 days (Fig. 1D).

To investigate the distribution of APP and A*ß* in platelets, we performed immunoelectron microscopy and found that APP was localized to platelet granule structures and at the plasma membrane of platelets. A*ß* was only marginally detected at the plasma membrane but not inside the cell (Fig. 1E). Statistical analysis of all images obtained from this experiment proved low detection of A*ß* (Fig. 1F).

### Platelets become Activated by Stimulation with Low and High Doses of Aß

**Figure 2:**
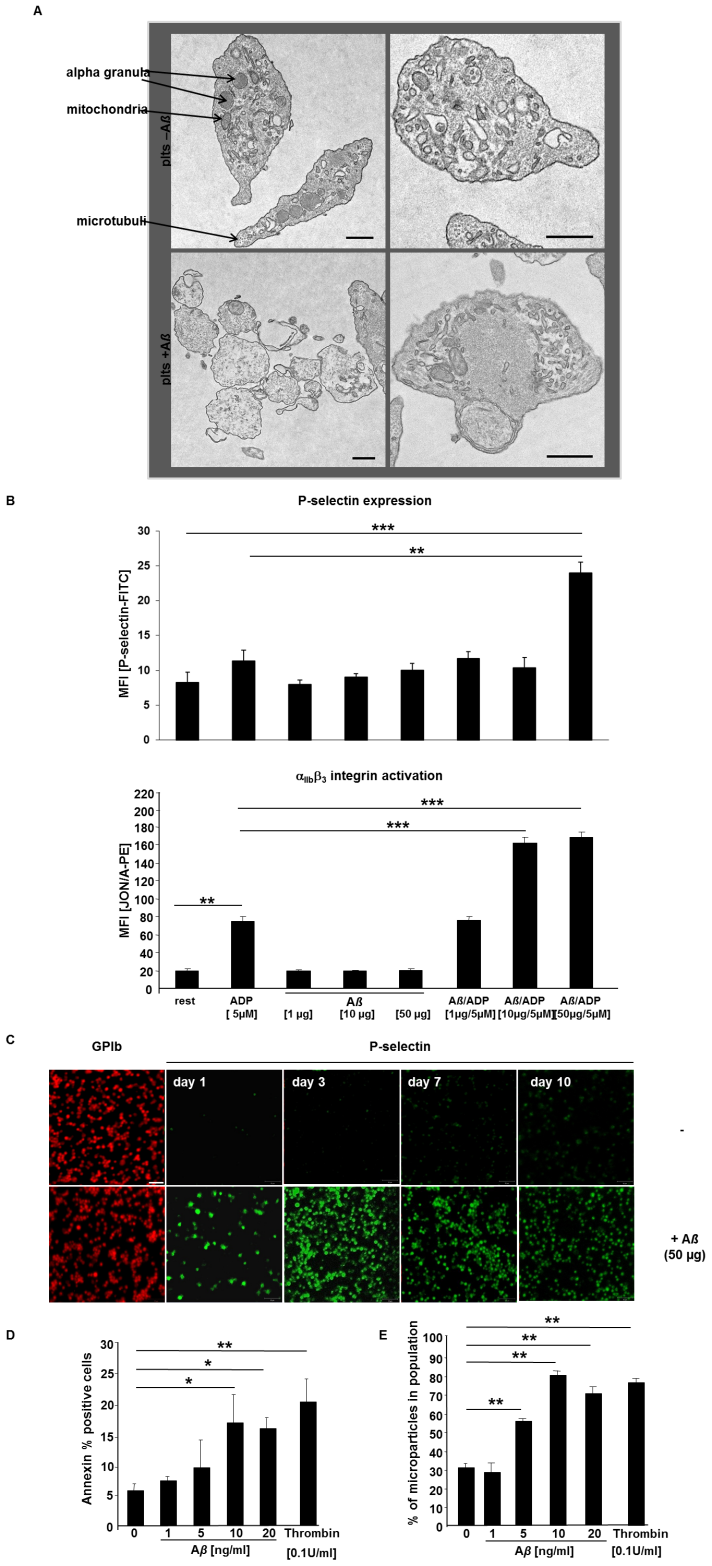
Stimulation of platelets with soluble A*ß* leads to platelet activation. (A) Platelets become activated by stimulation with 50 ng A*ß* (1–40) as shown by TEM (resting platelets upper panel, A*ß* stimulated platelets lower panel). Scale bar 5 µm. Intracellular organells were marked by arrows. (B) Blood was washed twice and incubated with 5 µM ADP, 1, 10 and 50 µg Aß and ADP/Aß as inidcated for 15 minutes in the presence of anti-P-selectin and JON/A-PE. Platelets were gated by their forward and side characteristics. The mean flourescence intensity (MFI) for each measurement is shown (n = 5 per group), **p<0.01 and ***p<0.001. (C) Platelets were isolated and stained for P-selectin as marker for platelet activation at indicated time points. (D) Arithmetic means ± SEM (n = 13) of the percentage of human platelets binding Annexin V Flous following a 60 min exposure to Tyrode buffer 2 mM CaCl_2_ in the absence (control) and presence of 1–20 ng/ml A*ß*. *p<0.05 and **p<0.01 indicates statistically significant difference to value in the absence of amyloid. (E) Arithmetic means ± SEM (n = 5) of the percentage microparticles from human platelets following a 60 min exposure to Tyrode buffer 2 mM CaCl_2_ in the absence (control) and presence of 1–20 ng/ml A*ß*. **p<0.01 indicates statistically significant difference to value in the absence of A*ß*.

To determine the impact of soluble A*ß* on platelet activation we first performed electron microscopy and confirmed morphological changes of platelets after incubation with A*ß* for 24 hrs. In contrast to the platelet suspension without stimulus, A*ß*-treated platelets showed typical characteristics of activated platelets such as transport of granules to the plasma membrane, concentration of actin, roundish morphology and already granule free cytoplasm possibly due to release of their α- and dense granule content (Fig. 2A). In addition we observed platelet aggregates after A*ß* stimulation (Fig. 2A).

Subsequent analysis of platelet degranulation (P-selectin exposure) and ß3-integrin activation (JON/A-binding) in response to A*ß* stimulation by flow cytometry revealed that A*ß* amplifies ADP-mediated platelet responses e.g. P-selectin expression (degranulation marker) and ß3-integrin activation (Fig. 2B) but is not sufficient to induce degranulation and integrin activation without additional stimulus suggesting that prolonged exposure of platelets to A*ß* might be required to induce full platelet activation without additional stimulus.

Determination of P-selectin expression in platelet cell culture with a singular treatment of soluble A*ß* over a time period of 10 days confirmed the results obtained from electron microscopy (Fig. 2C). We found P-selectin positive platelets over the whole time period with peak values at day 3 and 7 (Fig. 2C). Fluorescent annexin V was utilized to identify phosphatidylserine exposing platelets as indicator for coagulant activity of platelets [26]. As illustrated in Fig. 2D, exposure to A*ß* resulted in annexin V binding to the surface of platelets. The effect of A*ß* on annexin V binding reached statistical significance at ≥20 ng/ml A*ß* concentration. In addition, stimulation of platelets with A*ß* induced the formation of microparticles comparable to the extent of thrombin stimulation. Fig. 2E shows a significant increase in microparticle release after A*ß* stimulation (>5 ng/ml).

### Aß Stimulation Induces Platelet ROS Generation and Cell Membrane Scrambling

**Figure 3:**
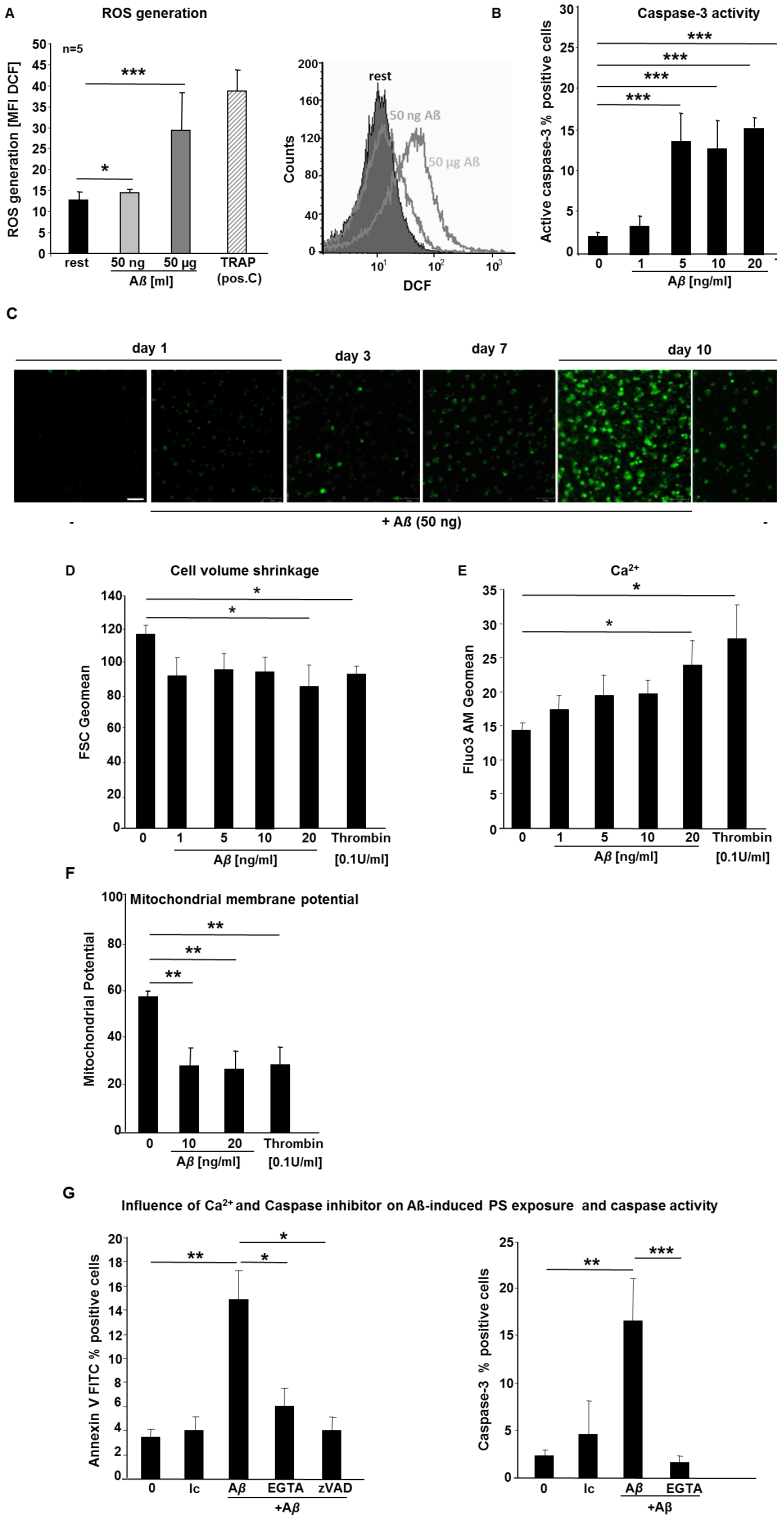
A*ß* stimulation of platelets induces ROS generation and cell membrane scrambling. (A) Generation of ROS from platelets is reported as mean fluorescence intensity (MFI) of DCF. Bar graphs depict mean values ±SEM (n = 5), *p<0.05 and ***p<0.001. TRAP stimulation of platelets was used as positive control. (B) Bar graphs depict mean values ±SEM (n = 13) of the percentage of human platelets expressing active caspase-3 following a 60 min exposure to Tyrode buffer (pH 7.4) 2 mM CaCl_2_ in the absence and presence of A*ß* (1–42) (1–20 ng/ml). ). *p<0.05 and ***p<0.001 indicates statistically significant difference to value in the absence of A*ß*. (C) A*ß* stimulated platelets were stained for caspase-3 at indicated time points and compared to non-stimulated platelets. (D). Bar graphs depict mean values ±SEM (n = 6) of the cell volume shrinkage in human platelets following a 60 min exposure to Tyrode buffer including 2 mM CaCl_2_ in the absence (control) and presence of 1–20 ng/ml A*ß*. *p<0.05 indicates statistically significant difference to value in the absence of amyloid. (E) Bar graphs depict mean values ± SEM (n = 7) of Fluo3AM fluorescence in FACS analysis reflecting calcium mobilization of platelets following a 60 min exposure to Tyrode buffer 2 mM CaCl_2_ in the absence and presence of A*ß* (1–20 ng/ml). *p<0.05 indicates statistically significant difference compared to control. (F) Bar graphs depict mean values ±SEM (n = 5) of DiOC_6_ fluorescence in FACS analysis reflecting mitochondrial membrane potential of platelets following a 60 min exposure to Tyrode buffer in the absence (control) and presence of A*ß* (1–20 ng/ml). **p<0.01 indicates statistically significant decrease in mitochondrial membrane potential (one-way ANOVA). (G) Bar graphs depict mean values ±SEM (n = 5, left panel) of the percentage of human platelets binding Annexin V-Fluos after pre-treatment with caspase inhibitor 1 µM zVAD-FMK (zVAD) followed by a 60 min. exposure with 20 ng/ml A*ß* in the presence of 2 mM CaCl_2_ and in the nominal absence of Ca^2+^ and presence of EGTA, **p<0.01 indicates statistically significant difference to the control in the absence of Aß, *p<0.05 indicates statistically significant difference to results with 20 ng/ml A*ß* alone in the absence of any pre-treatment. Bar graphs depict mean values ±SEM (n = 5, right panel) of the percentage of human platelets expressing caspase-3 after a 60 min exposure to 20 ng/ml A*ß* in the presence of 2 mM CaCl_2_ and in the nominal absence of Ca^2+^ and presence of EGTA, **p<0.01 indicates statistically significant difference to control experiments in the absence of A*ß*, ***p<0.001 indicates statistically significant difference to results with 20 ng/ml A*ß* alone in the absence of any pre-treatment. Ic = isotype control.

Reactive oxygen species (ROS) are chemically reactive molecules with important cell function in signaling and homeostasis [27]. ROS levels can increase upon environmental stress to control cellular function including apoptosis [28]. To investigate the impact of A*ß* in ROS generation of platelets we performed experiments using flow cytometry and found increasing ROS levels when platelets were stimulated with A*ß* (Fig. 3A). To promote removal of aged or activated platelets from the circulation, platelet senescence is associated with changes characteristic of apoptosis [29]. Caspases are central molecules of cell apoptosis and different studies showed that caspases also play a role in platelet activation and senescence [29]–[31].

Thus in additional experiments caspase-3 activity was analyzed by flow cytometry using platelets stimulated with increasing concentrations of A*ß*. As apparent from Fig. 3B exposure to A*ß* increased caspase-3 activity, an effect reaching statistical significance at ≥10 ng/ml A*ß* concentration. Furthermore, cell culture experiments showed caspase-3 positive platelets upon A*ß* stimulation over a time period of 10 days with peak values at day 10 (Fig. 3C).

Another hallmark of apoptosis is cell shrinkage. Thus cell volume was estimated from forward scatter in flow cytometry. As illustrated in Fig. 3D, A*ß* exposure was followed by a decrease of forward scatter reflecting cell shrinkage. Additional experiments aimed to identify mechanisms possibly involved in the stimulation of cell membrane scrambling by A*ß*. In a first series of experiments, cytosolic Ca^2+^ activity was estimated utilizing Fluo-3AM fluorescence. As illustrated in Fig. 3E, exposure of platelets to A*ß* increased Fluo-3AM fluorescence, an effect reaching statistical significance at 20 ng/ml A*ß* concentration. In further experiments mitochondrial cell membrane potential was determined utilizing DiOC_6_ fluorescence. As shown in Fig. 3F, exposure to A*ß* was followed by a decline of the mitochondrial potential, an effect reaching statistical significance at ≥10 ng/ml A*ß* concentration.

Additional experiments explored whether the observed alterations of cytosolic Ca^2+^ and of caspase-3 activity, contributed to or even accounted for the stimulation of cell membrane scrambling following A*ß* exposure. The impact of Ca^2+^ entry was explored by exposing platelets in the presence and absence of extracellular Ca^2+^ (addition of Ca^2+^ chelator EGTA instead of Ca^2+^). In a separate series of experiments platelets were pre-treated with pan-caspase inhibitor zVAD-FMK (1 µM) prior to A*ß* exposure. As shown in Fig. 3G (left panel), pre-treatment with the extra-cellular Ca^2+^ chelator EGTA or pan-caspase-3 inhibitor zVAD significantly blunted cell membrane scrambling by A*ß* treatment. Additional experiments elucidated the effect of Ca^2+^ removal on A*ß*-induced caspase-3 activation. As illustrated in Fig. 3G (right panel), the effect of A*ß* on caspase-3 activity was significantly blunted in the absence of Ca^2+^.

### Amyloid-ß Deposits in Platelet Cell Culture: Modulation of Soluble Aß Peptides by Platelets

**Figure 4:**
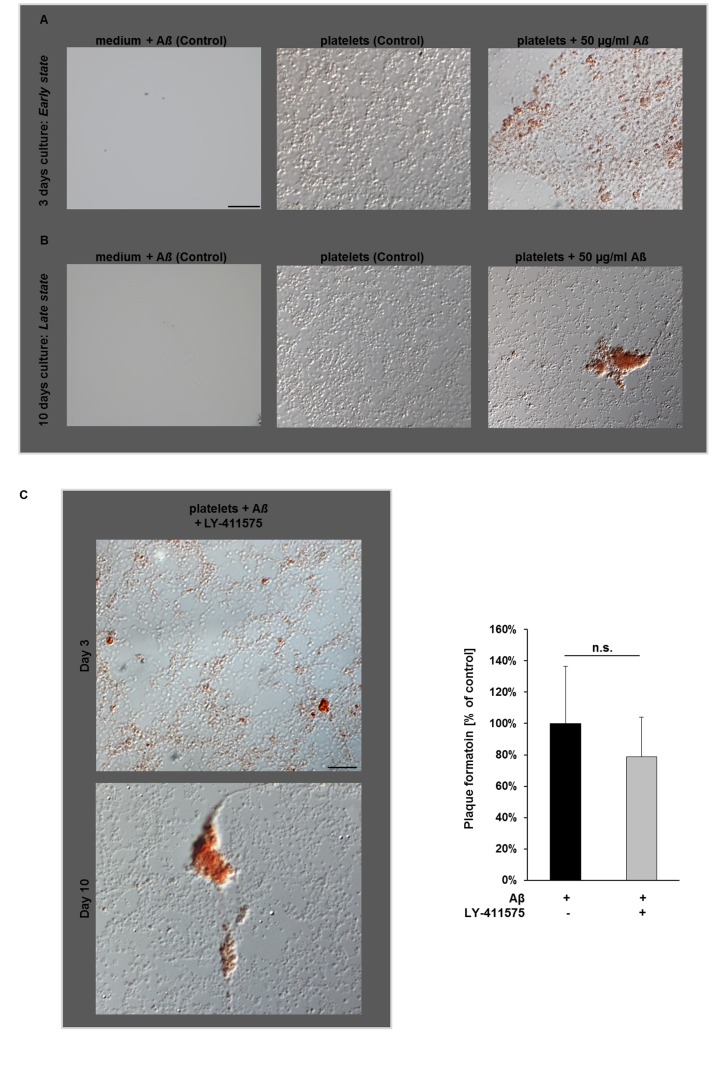
Formation of A*ß* deposits in human platelet cell culture. Congo red-positive platelets and A*ß* deposits in platelet cell culture after stimulation with 50 µg/ml A*ß* (A) after 3 days (*early state*, upper panel) and (B) after 10 days (*late state,* lower panel). Note the increase in extracellular A*ß* deposits after 10 days. Scale bar 20 µm. (C) Congo red-positive A*ß* aggregates are formed in the presence and absence of the APP inhibitor LY-411575 at indicated time points. Representative images (left) and bar graphs (right) are shown that depict mean values ±SEM (n = 6), n.s. = not significant. Scale bar 20 µm.

To further investigate, whether platelets are able to modify A*ß* peptides, we cultured platelets with soluble, synthetic A*ß* and analyzed modulation of A*ß* into amyloid fibrils by Congo red staining. Interestingly, we found Congo red-positive A*ß* deposits in platelet cell culture that were determined at different time points to analyze A*ß* aggregate formation by differential interference contrast (DIC) microscopy. As shown in Fig. 4A, platelets were congo red-positive after incubation with soluble synthetic A*ß* (50 µg/ml) for 3 days (*early state*). In addition, small extracellular fibrillar A*ß* aggregates were also detected.

When platelets were stimulated with soluble A*ß* for a longer time period (10 days, *late state*), large extracellular A*ß* deposits that were congo red-positive were determined by DIC microscopy suggesting, that either platelets were able to produce a great amount of A*ß* fibrils or that they have the ability to modulate soluble synthetic A*ß* (Fig. 4B). To test this hypothesis, we incubated platelets with synthetic A*ß* and the the γ-secretase inhibitor LY-411575 to avoid A*ß* production by platelets. Treatment of platelets with the γ-secretase inhibitor led again to congo red-positive A*ß* deposits comparable to those without inhibitor treatment (Fig. 4C). Thus, platelets are apparently able to modulate the soluble, synthetic A*ß* into fibrillar A*ß* that are deposited in platelet cell culture in a time- and concentration-dependent manner (Fig. 4 and Fig. S3). Using murine platelet cell culture we were able to confirm that also murine platelets are able to modulate soluble A*ß* peptides (Fig. S2).

### Incorporation of Extracellular, Synthetic A*ß* by Platelets Depends on Platelet Vitality

**Figure 5:**
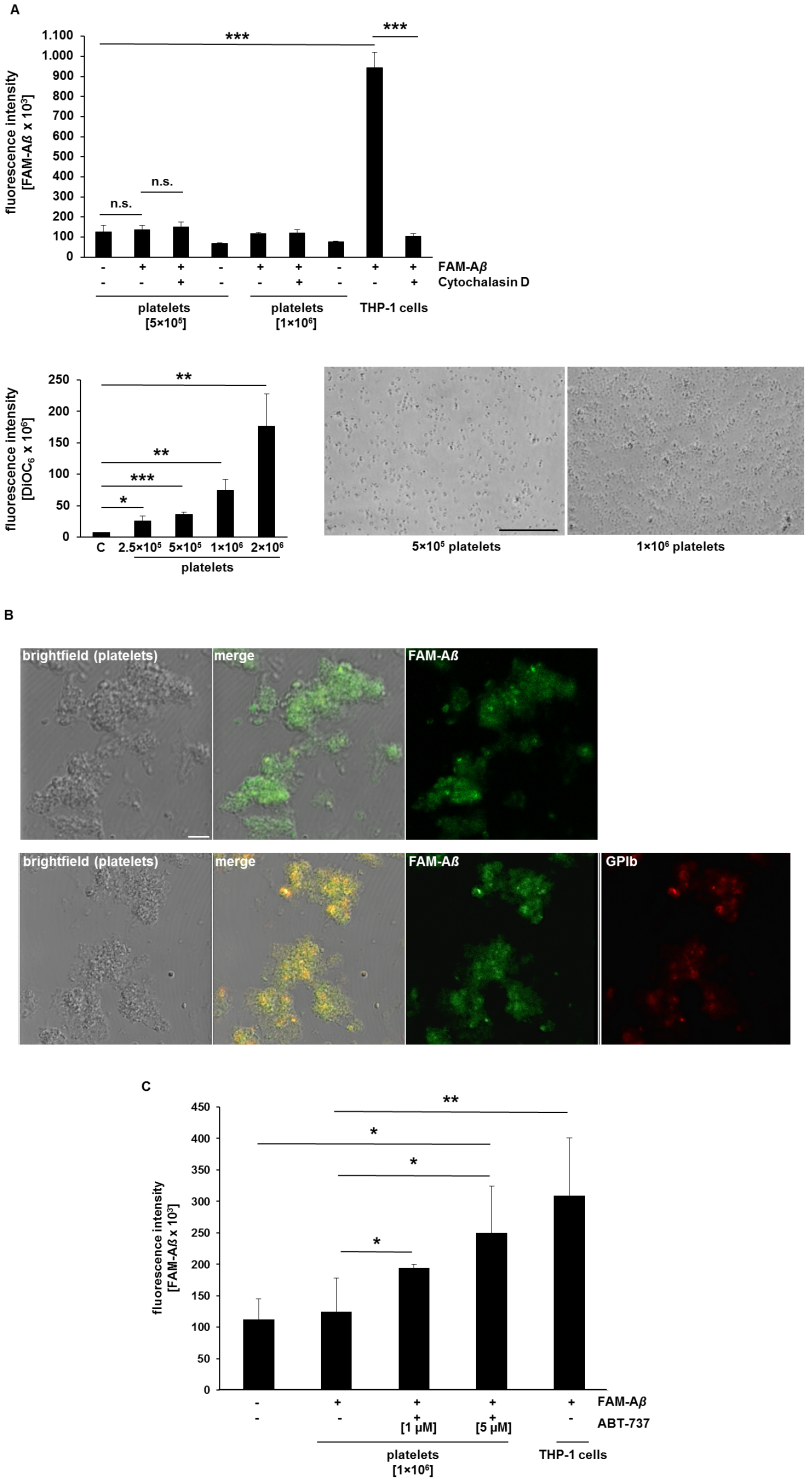
Incorporation of synthetic A*ß* fibrils by platelets. **(**A) Viable platelets do not absorb synthetic A*ß* fibrils. Different platelet concentrations were incubated with 0.5 µM pre-aggregated FAM-labeled A*ß* and fluorecsence intensity was measured (upper panel). 10 µM Cytochalasin D (Cyt D) was used as inhibitor of phagocytosis. THP-1 cells served as positive control. Presence of platelets was controlled by DiOC_6_ labeling (100 nM, lower left panel) and by phase contrast microscopy (lower right panel). N = 6; *p<0.05, **p<0.01 and ***p<0.001; n.s. = not significant. (B) Synthetic A*ß* was found on the plasma membrane of vital platelets as detected by confocal microscopy. FAM-A*ß* (green), platelet specific marker GPIb (red). Scale bar 20 µm. (C) Apoptotic platelets are able to incorporate pre-aggregated FAM-labeled A*ß*. Platelets were pre-treated with 1 and 5 µM ABT-737 to induce apoptosis. Fluorescence intensity of FAM-Aß was measured as indicated. Bar graphs depict mean values ±SEM (n = 6), *p<0.05, **p<0.01.

To analyze platelet mediated modulation of A*ß* in further detail, we tested, wether platelets are able to absorb A*ß* as observed for macrophages [32]. Thus, we incubated platelets with pre-aggregated FAM-labeled A*ß* for 4 hrs. and analyzed engulfment of labeled A*ß* by fluorescence intensity. As shown in Fig. 5A, viable platelets are not able to engulf A*ß* as seen for THP-1 cells which are known to phagocyte A*ß* and served as positive control (Fig. 5A). Furthermore, incubation of platelets with synthetic soluble A*ß* led to *Aß-coated platelets* that aggregated upon A*ß* stimulation (Fig. 5B). Co-staining with the platelet specific marker glycoprotein (GP)Ib confirmed localization of A*ß* at the plasma-membrane of platelets using confocal microscopy (Fig. 5B, lower panel).

Activation of apoptotic pathways in platelets can be induced by the pro-apoptotic agent ionomycin or ABT-737 leading to increased intraplatelet A*ß* accumulation [33]. Surprisingly, the stimulation of apoptotic pathways in platelets led to the incorporation of pre-aggregated synthetic A*ß* comparable to THP-1 cells known to phagocyte A*ß* (Fig. 5C). Taken together, vital platelets are not able to engulf A*ß* while the stimulation of apoptotic pathways in platelets enables them to engulf pre-aggregated A*ß*.

### Enhanced Platelet Adhesion upon A*ß* Stimulation of Platelets

**Figure 6:**
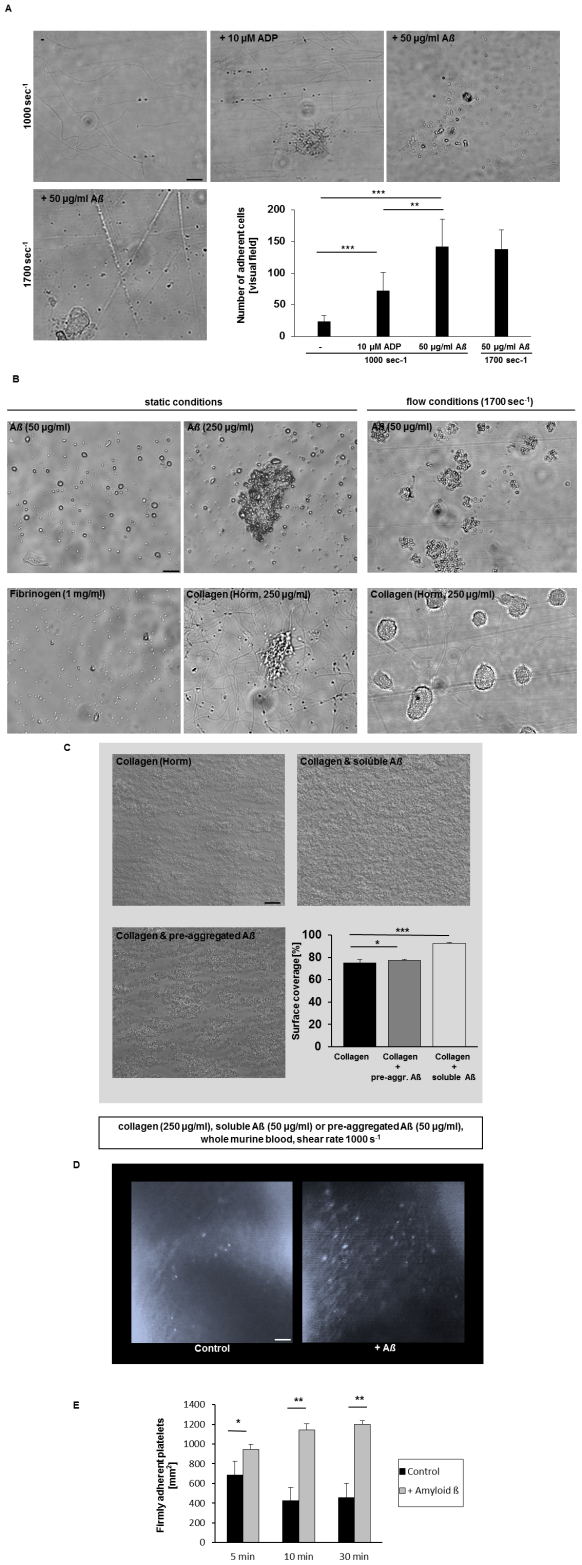
Strongly enhanced platelet adhesion under flow conditions *ex vivo* and on the injured carotid artery upon A*ß* stimulation of platelets *in vivo*. (A) Representative phase contrast images show platelet adhesion on collagen under flow conditions at a shear rate of 1000 sec^−1^ (upper panel) and 1700 sec^−1^ (lower panel) are shown. Scale bar indicates 20 µm. Bar graphs depict mean values ±SEM of the number of adherent cells per visual field [212×229 µm]. The platelet agonist ADP served as positive control, (n = 5 per group), **p<0.01 and ***p<0.001. (B) Platelets adhere to immobilized A*ß* under static and flow conditions (shear rate 1700 sec^−1^). Coverslips coated with 250 µg/ml collagen and 1 mg/ml fibrinogen served as positive control. Representative phase contrast images are shown. (C) Representative images show platelet thrombus formation on collagen and on collagen/A*ß*, respectively, at a shear rate of 1000 sec^−1^. Scale bar indicates 20 µm. Bar graphs depict mean values ±SEM of surface coverage, n = 5 per group, *p<0.05 and ***p<0.001. (D) Platelet adhesion at the injured carotid artery 10 min after injury. DCF-labeled platelets of C57BL/6J mice were incubated with vehicle or 50 µg/ml A*ß* for 30 min and injected into a C57BL/6J recipient mouse. Scale bar 50 µm. (E) Bar graphs depict mean values ±SEM showing the number of firmly adherent platelets at the vessel wall per mm^2^ after 5, 10 and 30 min after injury of seven independent experiments. *p<0.05, **p<0.01.

To elucidate the relevance of A*ß* for platelet adhesion under flow conditions, we perfused platelet-rich-plasma (PRP) over the extracellular matrix protein collagen that is exposed upon vessel injury. Pre-treatment of platelets with A*ß* led to enhanced platelet adhesion on collagen as seen for intermediate and high blood shear rates (24.14±9.25 cells/visual field versus 142.7±43.22 cells/visual field at a shear rate of 1.000 sec^−1^, Fig. 6A). Pre-treatment of platelets with ADP served as positive control. Furthermore, platelet adhesion was observed on immobilized A*ß* both under static and dynamic flow conditions (Fig. 6B). High amounts of immobilized A*ß* (250 µg/ml) induced platelet adhesion and aggregation (Fig. 6B, upper panel).

In further experiments we perfused whole blood over a mixed collagen/A*ß* matrix since accumulation of A*ß* in mice results into microbleeds because of vessel injury probably leading to exposure of extra cellular matrix proteins such as collagen. Here we were able to show enhanced accumulation and thrombus formation on a collagen/A*ß* surface compared to collagen alone (Figure 6C).

### Pre-incubation with Aß Strongly Enhances Platelet Adhesion at the Injured Carotid Artery of Mice

The above described results indicated that A*ß* stimulation of platelets results in platelet activation and induces apoptotic pathways characterized by mitochondrial depolarization, ROS generation, caspase activation and cell membrane scrambling. Furthermore, treatment of platelets with soluble A*ß* enhanced platelet adhesion to the extracellular matrix protein collagen. To test the significance of A*ß* mediated cellular changes of platelets in platelet accumulation at the injured vessel *in vivo*, we analyzed platelet adhesion at the injured carotid artery by *in vivo* fluorescence microscopy. To visualize and quantify platelet adhesion at the ligated carotid artery, platelets from donor mice were fluorescently labeled with DCF. Where indicated, platelets were pre-treated with 50 µg/ml soluble A*ß* prior injection into recipient mice. In contrast to control mice without A*ß* treatment, platelet adhesion at sites of injury was strongly enhanced when platelets were stimulated with A*ß* before injection into mice (Fig. 6D–E, video S1 and video S2) suggesting that A*ß* influences platelet activation and adhesion at injured vessels *in vivo*.

### Platelets are Recruited to Vascular Amyloid-ß Plaques in Brain of APP Dutch and APP23 Transgenic Mice

**Figure 7:**
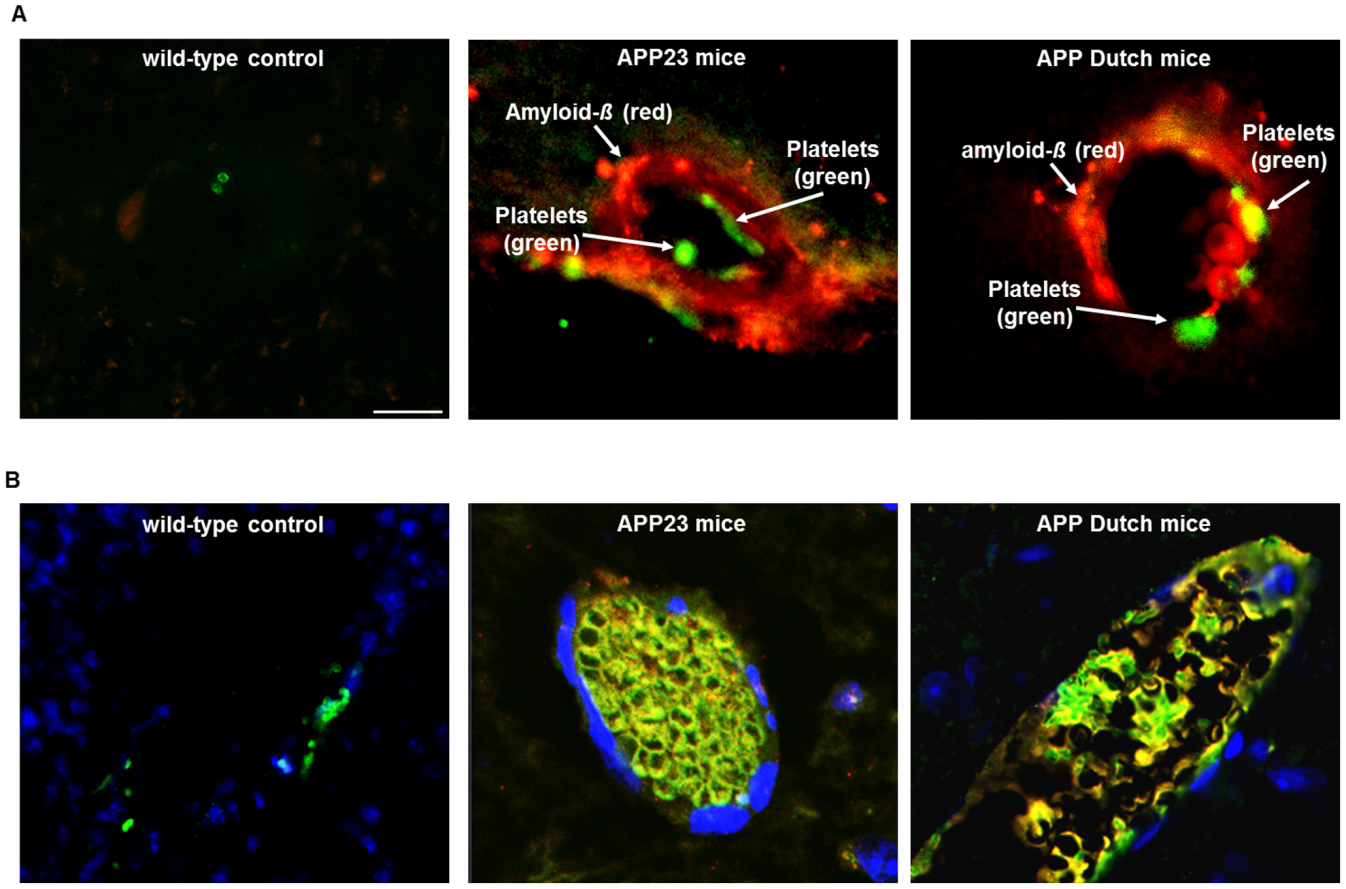
Recruitment of platelets to vascular amyloid-*ß* deposits in brain of APP Dutch and APP23 transgenic mice. (A) Platelet adhesion to vascular A*ß* plaques in cerebral vessels of both, APP23 (upper, middle panel) and APP Dutch (upper, right panel) transgenic mice was analyzed by confocal microscopy. Brains were immunohistochemically analyzed for A*ß* deposition (6E10, red) and the presence of platelets using the platelet specific marker GPIb (green). (B) Sustained platelet recruitment to vascular amyloid-*ß* deposits leads to full occlusion of the vessel (lower panel). Overlay of A*ß* immunoreactivity (red) and GPIb (green) is shown in yellow (merge), staining of cell nuclei (blue), scale bar 20 µm.

The use of control platelets and mice showed that treatment with A*ß* influences platelet physiology *in vitro* and *in vivo*. So far nothing is known about platelets in APP transgenic mice. APP Dutch and APP23 mice are known to develop CAA upon aging where amyloid-*ß* deposits localized mainly to the cerebral vessel walls. Thus we immunohistochemically analyzed brains of APP Dutch and APP23 mice for A*ß* deposition and platelet appearance. As shown in Fig. 7A, platelets adhere to vascular amyloid-*ß* plaques in the brain of both, APP Dutch and APP23 mice supporting the hypothesis of active platelet recruitment to amyloid plaques.

In addition, we found that sustained platelet recruitment to vascular amyloid-*ß* deposits can lead to full occlusion of the vessel. This suggests, that contact with A*ß* leads to platelet activation and production of A*ß* that amplifies platelet activation thus recruiting additional platelets from the blood stream resulting in full occlusion at sites of amyloid-*ß* deposits of cerebral vessels (Fig. 7B).

Taken together, A*ß* induces fundamental changes in platelet activation leading to uncontrolled platelet adhesion and aggregation *in* *vitro* and *in vivo*.

## Discussion

The present study reveals that contact of platelets with A*ß* leads to platelet A*ß* production, enhances platelet activation, triggers cell membrane scrambling and shrinkage of platelets, both hallmarks of apoptosis in other cell types. The effects are paralleled by ROS generation, increase of cytosolic Ca^2+^ activity, mitochondrial depolarization and activation of caspase-3. Furthermore we show that platelets modify soluble A*ß* leading to an increase in A*ß*_42_ in the supernatant and extracellular A*ß* aggregates/fibrils in a dose- and time-dependent manner in platelet cell culture. Whereas vital platelets are not able to absorb synthetic, pre-aggregated A*ß*, the induction of platelet apoptosis enables platelets to up-take A*ß* as shown for monocytic cells [32]. Interestingly, A*ß* stimulation of platelets enhances platelet adhesion under static and dynamic flow conditions *ex vivo* and at sites of vascular injury *in vivo* emphasizing our results from histological brain sections showing platelets localized to vascular amyloid-*ß* deposits in cerebral vessel walls of the AD transgenic mice APP Dutch and APP23. The concentrations required to achieve the observed effects are within the range of concentrations observed *in vivo*. However, it was shown, that plasma A*ß* levels fluctuated over time and differ between individuals, suggesting continuous contributions from brain and peripheral tissues and association with reactive circulating proteins [17]. Concentrations of A*ß* were reported to approach 150 pg/ml in plasma, 6100 ng/gm in brain grey matter, 1100 ng/gm in brain white matter, 1 ng/ml in minimal atherosclerotic lesion in arteries and 28 ng/gm in leptomeningial arteries with atherosclerotic plaques [17].

The release of A*ß* peptides by circulating platelets was shown by different groups in the past using different platelet agonists. Already in the late nineties, A*ß* release from platelets was detected upon stimulation with collagen [34] and thrombin [35]. In contrast we found amounts of released A*ß* upon A*ß* stimulation that were >10 times higher compared to stimulation with platelet agonists. Surprisingly, treatment with the γ-secretase inhibitor LY 411575 revealed that the major amount of A*ß*_42_ was rather generated by platelet-mediated modification of soluble A*ß*_40_ than produced by platelets.

Smirnov and colleagues provided evidence that thrombin and prostaglandin 2 regulate the processing and secretion of A*ß* peptides and sAPP in human platelets [23]. Here we show for the first time that platelets release A*ß* in a dose- and time-dependent manner when they come into contact with soluble exogenous A*ß* that is also present in blood under physiological and pathophysiological conditions [17]. Furthermore, we detected A*ß* in platelet lysates that may arise from A*ß* processed at the plasma membrane of platelets. In contrast we only marginally detected A*ß* at the plasma membrane by immunoelectron microscopy. However, there are some limitations of the immunogold technique that makes localization of weak A*ß* staining in platelets difficult. Gold particles are around 15–30 nm away from the site to which the primary antibody is bound, thus the precise localization of A*ß* cannot be accurately calculated [36]. Furthermore, ultra-thin sections (30 nm) are made for immunogold labelling that can produce misleading results because a thin section of cells may not give an precise view of its three-dimensional structure [36]. In particular, platelets display invaginations of the plasma membrane that can also account for misleading results of localized A*ß*. Therefore it seems as if A*ß* is also found intracellular and not only at the cell surface.

The fact that A*ß* can be released by platelets strengthens the hypothesis that the origin of A*ß* in blood might be an (additional) release of A*ß* from blood cells and other non-neuronal cells [37] while others believe that circulating A*ß* could derive from the central nervous system through the blood brain barrier [38].

The effects of amyloid on platelets apparently involves platelet activation e.g. platelet aggregation as already shown by Shen and colleagues [20], [39]. Application of exogenous A*ß* (1–2 µM) potentiated platelet aggregation mediated by collagen and other agonists while at higher concentrations (5–10 µM), A*ß* induced platelet aggregation without additional stimulus. In contrast to this study we were not able to detect typical parameters of platelet activation such as P-selectin exposure and integrin activation even with high doses of A*ß* alone (50 µg, 11.5 µM). A*ß* was shown to amplify platelet responses induced by the weak platelet agonist ADP. However, Annexin V positive cells and microparticle formation were already detected upon stimulation with low doses of A*ß* demonstrating that variable concentrations of A*ß* are needed for different processes of platelet activation.

The generation of reactive oxygen species, defects in mitochondrial function and amyloid metabolism have been already implicated in the pathophysiology of AD in the late nineties [40]. Here we show for the first time that contact of platelets with A*ß* induces platelet-mediated ROS production and declines mitochondrial potential that might account for increased ROS levels in brain. Furthermore, the effects of A*ß* on platelets apparently involve activation of caspases, as it is significantly blunted in the presence of pancaspase inhibitor zVAD-FMK. The amyloid-induced stimulation of caspase-3 and cell membrane scrambling results at least in part from Ca^2+^ entry, as it is almost abolished by removal of extracellular Ca^2+^. The presence of Ca^2+^ is further a prerequisite for the stimulation of caspase-3 and of cell membrane scrambling. Beside caspase-3 activity and ROS generation, we found other hallmarks of apoptosis and cell membrane scrambling in platelets after contact with A*ß* such as cell shrinkage suggesting that A*ß* induces apoptosis in platelets. Zaghi and co-workers already provided strong evidence that blood cells such as macrophages underwent apoptosis upon exposure to soluble or fibrillar Aß [32]. More important macrophage apoptosis alters the ability of these cells to ingest and clear A*ß* [32]. This proposes that enhanced A*ß*-induced platelet apoptosis also alters blood homeostasis and clearance of platelets from circulating blood as a result of an increase in phosphatidylserine exposing platelets that are engulfed by phagocytosing cells. Accordingly, excessive platelet apoptosis could thus cause thrombocytopenia. Amyloidosis may indeed be complicated by thrombocytopenia [41].

Beside A*ß*-mediated activation and apoptosis of platelets and A*ß* release from activated platelets we provided strong evidence for platelet-mediated modulation of soluble A*ß* showing that platelets modulate A*ß*_42/40_ ratio as well as soluble exogenously applied synthetic A*ß*_40_ and congo-red positive fibrillar A*ß* deposits in a time- and concentration-dependent manner that might contribute to A*ß* plaque formation at cerebral vessels detected by immunohistology of brain sections of APP Dutch and APP23 transgenic mice. Experiments using the APP inhibitor LY-411575 confirmed that A*ß* aggregates/fibrils in platelet cell culture are not a result of Aß_40/42_ release of platelets but may develop from modulation of exogenously applied synthetic A*ß*. It is tempting to speculate how platelets are able to achieve modulation of A*ß*. Different studies in the past already detected membrane abnormalities in platelets of AD patients [42]–[44]. A more recent study provided evidence for a modification of membrane properties of platelets from AD patients produced by increased expression and activity of the nitrergic system [45]. If these alterations in membrane properties of platelets in AD patients have any impact in the modulation of soluble A*ß* must be analyzed in future experiments. The contradicting results emerged from ELISA measurements of A*ß* versus modified A*ß* which has been added to the cell culture may be due to the fact that A*ß* is only partially modified to fibrillar congo-red positive A*ß* while another part is still soluble and thus can be measured by ELISA. However, we do not know the composition of fibrillar A*ß* molecules that are produced by platelet-mediated modification in cell culture.

Platelets are not able to engulf A*ß*, neither in its soluble nor in a pre-aggregated form as observed for macrophages [32], [46]. In contrast, induction of apoptotic pathways in platelets by ABT-737 led to uptake of pre-aggregated A*ß* into platelets. A significant increase in intraplatelet Aß_42_ was already shown for platelets stimulated with ionomycin [33], leading to the hypothesis that apoptosis of platelets might be related to altered processing of APP [2]. We hypothesize that uptake of A*ß* into pro-apoptotic platelets might enhance local accumulation of A*ß* when platelets adhere on vascular amyloid plaques at cerebral vessels and undergo apoptosis thereby enhancing cerebral amyloid angiopathy. However, macrophages are known to phagocyte and clear A*ß*, processes that were altered in AD patients showing less clearance of A*ß* but enhanced apoptosis upon exposure to A*ß*. The authors conclude that macrophages shuttle A*ß* from neurons to vessels where they undergo apoptosis and subsequently release fibrillar A*ß* contributing to CAA [32]. Other proposed mechanisms for the pathogenesis of CAA include A*ß* production by myocytes in the vessel wall [47] and more recently A*ß* deposition from interstitial fluid being drained from the central nervous system [48].

In this study we provided strong evidence that A*ß* activation of platelets initiates a cycle of A*ß* release and consequently A*ß* mediated platelet activation followed by enhanced platelet adhesion on subendothelial matrix proteins such as collagen under flow conditions as well as at the injured carotid artery as shown here for the first time *ex vivo* and *in vivo*. In addition, we show that platelets can adhere to immobilized A*ß* under flow to form platelet aggregates, a fact that was further supported by immunohistology studies demonstrating platelet adhesion on A*ß* deposits in cerebral vessel walls of AD transgenic mice known to develop CAA. Furthermore, these results are supported by experiments showing that platelets are able to modulate soluble A*ß* into fribrillar structures that all might contribute to CAA. More critically continued platelet recruitment to A*ß* deposits can lead to full occlusion of the vessel, emphasizing the above described hypothesized cycle of A*ß*-mediated activation of platelets that get into contact with A*ß* followed by A*ß* release that amplifies platelet activation to recruit additional platelets from the blood stream leading to full occlusion of cerebral vessels suggesting micro-embolization events in AD transgenic mice that might lead to thrombotic events like stroke.

The association of stroke and Alzheimer disease was already suggested for human individuals where the relation is strongest in the presence of vascular risk factors while it remained weakly in the absence of these factors [4]. Results from a case control study of human brain biopsies indicates that CAA is a risk factor for ischemic cerebral infarction [49]. In addition, MRI studies provided evidence that advanced CAA predisposes to ischemic infarction as well as intracerebral haemorrhage [50]. More interesting, Schneider and colleagues hypothesized that subcortical infarcts contribute to the destructive effects of A*ß* plaque formation in AD pathology by increasing dementia and lowering memory function [51]. However, to date no clear evidence exists that confirmed the correlation of AD and stroke leading to declined cognitive function in APP transgenic mice and patients.

In conclusion, treatment of platelets with A*ß* is followed by platelet activation, platelet A*ß* release, ROS generation, increase of cytosolic Ca^2+^ activity, mitochondrial depolarization, activation of caspase-3, cell shrinkage and cell membrane scrambling. Furthermore, our results point to a new role for platelets as major contributors to CAA caused by different mechanisms such as A*ß* release, modulation of A*ß* from A*ß*_40_ into A*ß*_42_ and soluble to fibrillar structure, A*ß* up-take in apoptotic platelets and adhesion and accumulation of platelets at amyloid-*ß* deposits of cerebral vessels leading to vessel occlusion and microembolization to potentiate the risk of stroke under AD pathology.

## Materials and Methods

### Chemicals and Antibodies

Platelets were activated by ADP (Sigma-Aldrich) and soluble Amyloid *ß* (1–40), American Peptide, Cat. No. 62-0-78A, Sequence Asp-Ala-Glu-Phe-Arg-His-Asp-Ser-Gly-Tyr-Glu-Val-His-His-Gln-Lys-Leu-Val-Phe-Phe-Ala-Glu-Asp-Val-Gly-Ser-Asn-Lys-Gly-Ala-Ile-Ile-Gly-Leu-Met-Val-Gly-Gly-Val-Val. A stock solution with a concentration of 1 mg/ml was prepared using Amyloid *ß* (1–40) solved in sterile Tris buffer and stored at −20°C. Antibodies against APP C-terminal fragment (Covance SIG-39152), amyloid-*ß* (1–16) (Covance, 6E10, SIG-39300) and CN3 [52] were used for immunohistochemistry and immunogold Electron Microscopy. Fluorophore-labeled antibodies anti-P-selectin-FITC (Wug.E9-FITC, Rat IgG2b, Emfret Analytics) and anti-integrin αIIbβ3-PE (JON/A-PE, Rat IgG1, Emfret Analytics) were used for flow cytometric analysis.

### Animals

Specific pathogen-free C57BL/6J mice were obtained from Charles River (Sulzfeld, Germany). All animal experiments were conducted according to the Declaration of Helsinki and German law for the welfare of animals. The protocol was approved by the Heinrich Heine University Animal Care Committee and by the district government of North Rhine-Westphalia (LANUV, NRW; Permit Number: 84-02.05.20.12.284; O 86/12; 84-02.04.2012.A405).

### Isolation and Stimulation of Human Platelets

Fresh ACD-anticoagulated blood was obtained from healthy volunteers between the age of 22 to 50 years. Participants provided their written informed consent to participate in this study according to the Ethics Committee of the Eberhard Karl University Tuebingen, Germany (184/2003 V). To document the process, age and gender were noted. The Ethics Committee of the Eberhard Karl University Tuebingen, Germany approved the consent procedure and specifically this study according to the Declaration of Helsinki. The blood was centrifuged at 200 g for 20 minutes at 25°C without brake. The platelet rich plasma was separated, added with Tyrode buffer (137 mM NaCl, 2.8 mM KCL, 12 mM NaHCO3, 5 mM glucose, 0.4 mM Na2HPO4, 10 mM HEPES, 0.1% BSA), pH 6.5 in 1∶7 volumetric ratio and centrifuged at 900 g and 25°C for 10 minutes with brake. The platelet pellet was resuspended in 500 µl Tyrode buffer pH 7.0.

### Murine Platelet Preparation

Platelets were prepared as previously described [53], [54]. Briefly, murine blood from retro-orbital plexus was collected and centrifuged at 1800 rpm for 5 minutes at room temperature. To obtain platelet-rich plasma (PRP), the supernatant was centrifuged at 800 rpm for 6 min. PRP was washed twice at 2800 rpm for 5 min at room temperature and pellet was resuspended in Tyrode’s buffer [136 mM NaCl, 0.4 mM Na2HPO4, 2.7 mM KCl, 12 mM NaHCO3, 0.1% glucose, 0.35% bovine serum albumin (BSA), pH 7.4] supplemented with prostacyclin (0.5 µM) and apyrase (0.02 U/mL). Before use, platelets were resuspended in the same buffer and incubated at 37°C for 30 min.

### Stimulation of Platelets

Platelets in a concentration of 10^6^/ml were stimulated in Tyrode’s buffer (pH 7.4) containing 2 mM CaCl_2_. Where indicated, A*ß* was as added at the indicated concentrations (1, 5, 10, 20, 50 ng/ml and 50 µg/ml) for 60 minutes at 37°C. A negative control without amyloid and a positive control with thrombin 0.1 U/ml were analyzed simultaneously with each set of experiment.

### Human and Murine Platelet Culture

Human or murine platelets were prepared as described and adjusted to a final concentration of 2×10^7^ platelets per 150 µl medium (DMEM). Platelets were stimulated with 50 ng or 50 µg A*ß* for indicated time points. After incubation, unbound platelets were removed by rinsing with Tyrode’s buffer while adherent platelets were fixed with 4% paraformaldehyde and stained for A*ß*, caspase-3 and P-selectin, respectively. Samples were analyzed by confocal microscopy and at least 8 confocal images were taken for each sample. For the analysis of amyloid-*ß* plaque formation, Congo red-positive A*ß* deposits in platelet cell culture were determined at different time points. Where indicated, platelets were pre-treated with the γ-secretase inhibitor LY 411575 (0.1 µM). Analysis was performed using a 63x objective and a Zeiss Axioskop 2 microscope (Zeiss).

### Amyloid ß Quantification by Enzyme Lnked Immunosorbent Assay (ELISA)

Platelets were adjusted to a final concentration of 2×10^7^ in a volume of 150 µl and stimulated with Amyloid ß as indicated. Accumulation and release of A*ß* into the platelet cell supernatant was measured for the indicated time points following the manufacturer’s protocol (Aβ 42 Human ELISA Kit, Invitrogen). Where indicated, platelets were pre-treated with the γ-secretase inhibitor LY 411575 (0.1 µM).

### Flow Cytometry

Flow cytometry analysis was performed as described elsewhere [55]. Briefly, two-colour analysis of murine platelet activation was performed using fluorophore-labeled antibodies for P-selectin expression (Wug.E9-FITC) and the active form of αIIbβ3 integrin (JON/A-PE). Heparinized blood was diluted in Tyrode buffer and washed twice. Blood samples were mixed with antibodies after addition of 1 mM CaCl2 and stimulated with indicated agonists for 15 min at room temperature. Reaction was stopped by the addition of PBS and samples were analyzed on a FACSCalibur flow cytometer (BD Biosciences).

### Transmission Electron Microscopy (TEM)

Resting or activated platelets were incubated with A*ß* in medium for 24 hrs and controlled by phase contrast microscopy. Subsequently, the cells were fixed in Karnovsky’s solution for 1 h at room temperature and stored at 4°C. For electron microscopic studies, cell pellets were embedded in agarose at 37°C, coagulated, cut in small blocks, fixed in Karnovsky’s solutions, postfixed in osmium tetroxide, and embedded in glycid ether and cut using an ultramicrotome (Ultracut Reichert, Vienna, Austria). Ultrathin sections (30 nm) were mounted on copper grids and analyzed using a Zeiss LIBRA 120 transmission electron microscope (Carl Zeiss, Oberkochen, Germany) [56]–[58].

### Immunogold Electron Microscopy (IEM)

For IEM of A*ß* and the precursor APP, platelets were fixed in glutaraldehyde (0.01%) and paraformaldehyde (3%) and embedded in 3% agarose. Small parts of the agarose blocks were embedded in Lowicryl (Polysciences Ltd.). 30-nm ultra-thin sections were mounted on formvar-coated nickel grids and incubated with mouse APP-C-Term antibody and A*ß* antibody, respectively, followed by 12 nm gold-conjugated goat anti–mouse IgG1 (Jackson ImmunoResearch, Dianova, Germany). In control samples, platelets without A*ß* were used. Grids were counterstained with uranyl acetate and examined using a transmission electron microscope (Zeiss LIPRA 120) [59], [60].

### Phagocytosis Assay

For the analysis of the phagocytosis potential of platelets, synthetic Aß was prepared as follows: FAM-labeled A*ß* (Anaspec, Beta-Amyloid (1–42)-Lys(Biotin), FAM–labeled, Sequence: FAM-Asp-Ala-Glu-Phe-Arg-His-Asp-Ser-Gly-Tyr-Glu-Val-His-His-Gln-Lys-Leu-Val-Phe-Phe-Ala-Glu-Asp-Val-Gly-Ser-Asn-Lys-Gly-Ala-Ile-Ile-Gly-Leu-Met-Val-Gly-Gly-Val- Val-Ile-Ala-Lys(Biotin)-OH) was dissolved at equimolar concentrations in PBS to reach a final concentration of 15 µM total Aß. To generate fibrils, the peptide was incubated for 3 days at 37°C and subsequently stored at −20°C. Platelets (1×10^6^) were seeded in a volume of 150 µl medium in 96 well plates. FAM-labeled synthetic A*ß* preparation was added and incubated for 4 hrs. under normal growth conditions. Medium was aspirated and 100 µl trypan blue solution was added to quench extracellular fluorescence. After removing trypan blue, intracellular fluorescence was measured at 485 nm (excitation) and 535 nm (emission). To control the presence of cells, platelets were labeled with 100 nM DIOC_6_ and additionally monitored by phase contrast microscopy.

### Phosphatidylserine Exposure

Phosphatidylserine exposure resulting from cell membrane scrambling was measured after stimulation with A*ß*, followed by centrifugation of cells at 1000 g for 2 minutes and incubation with 1∶20 dilution of Annexin V Fluos (Immunotools, Germany) in Tyrode buffer (pH 7.4) supplemented with 2 mM CaCl_2_ for 30 minutes in the dark at room temperature. The forward scatter was determined and the fluorescence was measured in FL-1 of a BD FACSCalibur (BD Biosciences, CA, USA).

### Cell Volume and Microparticle Release

Isolated platelets were resuspended at a concentration of 10^6^/ml of Tyrode buffer (pH 7.4) with 2 mM CaCl_2_ and stimulated with designated concentrations of A*ß* (1–20 ng/ml) for 60 minutes at 37°C and washed once in Tyrode buffer (pH 7.4). Washed platelets were stained with GPIb (CD42b PE, BD Biosciences) for 30 minutes at room temperature in the dark and analyzed in the FSC vs. SSC plot with FSC (E00) settings in the Log scale for cell volume (FCS Geomena) and E01 for detection of microparticles [61].

### Cytosolic Calcium

Intracellular Ca^2+^ concentration was measured following stimulation with A*ß* as indicated, washing once in Tyrode buffer (pH 7.4) with 2 mM CaCl_2_, staining with 5 µM Fluo-3AM (Biotium, USA) in the same buffer and incubating at 37°C for 30 minutes. The fluorescence was measured in FL-1 of a BD FACSCalibur (BD Biosciences, CA, USA).

### Caspase-3 Activity

For determination of caspase-3 activity, 10^6^ platelets/ml were suspended in Tyrode buffer (pH 7.4) with 2 mM CaCl_2_ and stimulated with 1, 5, 10 and 15 ng/ml concentrations of amyloid. Caspase-3 activity was measured by CaspGlow Fluorescein Active Caspase-3 Staining kit from BioVision (CA, USA) as per the manufacturer’s instruction and fluorescence intensity was measured in FL-1 in BD FACSCalibur (BD Biosciences, USA).

### Mitochondrial Membrane Potential

In order to measure the depolarization of the outer membrane of platelet mitochondria, 10^7^ platelet/ml of Tyrode buffer (pH 7.4) with 2 mM CaCl_2_ were first stimulated with A*ß* as described before. Then platelets were centrifuged and resuspended in phosphate buffered saline (PBS) (Invitrogen, CA, USA) supplemented with 1 mM MgCl_2_, 5.6 mM glucose, 0.1% BSA and 10 mM HEPES (pH 7.4) in a total volume of 1 ml and stained with 10 nM DiOC_6_ (Invitrogen, CA, USA) for 10 minutes. The stained cells were centrifuged at 1000 g for 2 minutes at 25°C, resuspended in PBS and measured by flow cytometry [62].

### Free Radical Generation (ROS) from Platelets in Response to A*ß*

The intracellular ROS generation of cells can be investigated using the 2′,7′-dichlorofluorescein-diacetate (DCFH-DA). Platelets (2×10^6^/ml) were incubated in the presence of unlabeled A*ß* (50 ng/ml and 50 ug/ml) for 24 hrs. Following which platelets were treated with DCFH-DA (10 µM) and mouse anti-human GPIb (CD42b-PE, Beckman Coulter) antibody for 30 minutes at room temperature. Resting platelets were treated with platelet agonist TRAP (25 µM) in the presence of DCFH-DA and mouse anti human CD42b-PE to compare ROS generation among A*ß* treated and agonist activated platelets with respect to non-activated resting platelets. At the end of the incubation period samples were analyzed by flow cytometry (FACS Calibur, Becton Dickinson) gating for the platelet specific CD42b positive population. Fluorescence of 10.000 cells was acquired and analyzed by the Cell Quest program to determine the mean fluorescence. Generation of ROS from platelets is reported as mean fluorescence intensity of DCF [63].

### Platelet Adhesion under Flow using the Flow Chamber

Cover slips (24×60 mm) were coated with 200 µg/mL fibrillar type I collagen (Nycomed) or human fibrinogen (1 mg/ml) overnight and then blocked with 300 µl of 1% BSA solution for at least 60 min. Tyrode’s buffer was prepared as described before. PH was adjusted to 7.4 and the Tyrode’s buffer was prewarmed at 37°C. Mice were anesthetized with isoflurane and blood was taken from the retro-orbital plexus of each mouse and collected in a tube containing 300 µL Heparin (20 U/ml in TBS). PRP was prepared as described and pre-incubated with ADP (positive control) or A*ß* at 37°C and put in a 1 ml syringe. Platelets were perfused through the flow chamber at desired shear stress of 1000 sec^−1^ or 1700 sec^−1^ and platelet adhesion and aggregate formation was evaluated.

### Platelet Adhesion on Immobilized Amyloid ß

Synthetic A*ß* (50 and 250 µg/ml) was immobilized on cover slips (24×60 mm) overnight and then blocked with 300 µl of 1% BSA solution for at least 60 min. PRP was prepared as described and platelet count was adjusted to 2×10^8^ platelets/ml. Platelet adhesion and aggregate formation was analyzed under static and flow condition. In additional experiments, whole blood was perfused over a collagen/A*ß* matrix and thrombus formation was analyzed by surface coverage.

### Carotid Ligation in Mice and Assessment of Platelet Adhesion by Intravital Microscopy

To evaluate the effect of A*ß* on platelet activation and adhesion *in vivo*, we used intravital fluorescence microscopy. Prior to the experiments, platelets from donor mice were stained with 5-carboxyfluorescein diacetate succinimidyl ester (DCF) and incubated with 50 µg/ml soluble A*ß* for 30 min. Wild-type C57BL/6J mice (Charles River Laboratories) were anesthetized with medetomidine, midazolame and fentanyl. Polyethylene catheters (Portex) were implanted into the right jugular vein, and fluorescent platelets (8×10^6^/150 µL) were injected intravenously [53], [55], [64]. The common carotid artery was dissected free and ligated vigorously for 5 min to induce vascular injury. Before and after vascular injury, interaction of the fluorescent platelets with the injured vessel wall was visualized by *in vivo* video microscopy of the common carotid artery using a Leica microscope (20×water immersion objective, W 20×/0,5; Leica DM2500MH) [53], [55], [64].

### Histology and Immunohistochemistry

Brains were removed and immersion fixed for 48 hrs in paraformaldehyde (4%), then cryoprotected in 30% sucrose for additional 2 days [1]. After freezing, serial 25-µm thick coronal sections were cut through the brains using a freezing-sliding microtome. The sections were collected in 0.1 M Tris-buffered saline and stained immunohistochemically. Polyclonal antibody CN3 [52] was used for immunostaining of A*ß* and monoclonal antibody CD42b was used for immunostaining of the platelet specific glycoprotein Ib (GPIb) (US Biological).

### Statistical Analysis

Data are provided as arithmetic means ± SEM (*standard error of mean*), statistical analysis was made by one-way ANOVA or student’s paired t-test, where applicable.

## Supporting Information

Figure S1 **Accumulation of A** ***ß*** **(1–42) measured by a sandwich ELISA assay.** A*ß* (1–42) levels measured in different control experiments: Medium alone, supernatant of resting platelets and medium supplemented with 50 ng/ml Aß (1–40) and 50 µg/ml Aß (1–40), respectively. Bar graphs depict mean values ±SEM (n = 4–8) of Aß1–42 levels as indicated.
(PDF)

Figure S2 **Formation of A** ***ß*** **deposits in murine platelet cell culture.** Congo red-positive platelets and A*ß* deposits in platelet cell culture of C57BL/6J mice after stimulation with 50 µg/ml A*ß* in the presence and absence of the APP inhibitor LY-411575 after 10 days in culture. (right panel). Approaches with medium and A*ß* or platelets alone served as controls to confirm that A*ß* does not pre-aggregate to produce congo red-positive A*ß* deposits spontaneously. Scale bar 20 µm.
(PDF)

Figure S3 **Platelet-mediated modulation of soluble A** ***ß*** **into congo red-positive fibrils is concentration-dependent.** Congo red-positive platelets and A*ß* deposits in platelet cell culture after stimulation with indicated concentrations of soluble A*ß* after 10 days. Note the increase in extracellular A*ß* deposits with Aß concentrations >1 µM. Scale bar 20 µm.
(PDF)

Video S1 **Video of** ***in vivo*** **platelet adhesion at the injured carotid artery.** Platelet adhesion to the ligated carotid artery was analyzed by intravital fluorescence microscopy using platelets from donor mice without any (pre-) treatment (control mouse). Platelets were labeled with DCF for visualization and quantification.
(AVI)

Video S2 **Video of** ***in vivo*** **platelet adhesion at the injured carotid artery after pre-treatment with A** ***ß*** **.** Video S2 shows enhanced platelet adhesion to sites of injury after pre-treatment of donor platelets with A*ß* prior injection into recipient mouse. Platelets are labeled with DCF for visualization and quantification.
(AVI)
