# Supplementary figures and images for: Blood Platelets in the Progression of Alzheimer’s Disease
Source: PLoS One. 2014 Feb 28;9(2):e90523. doi: 10.1371/journal.pone.0090523 (PMC3938776; doi:10.1371/journal.pone.0090523)

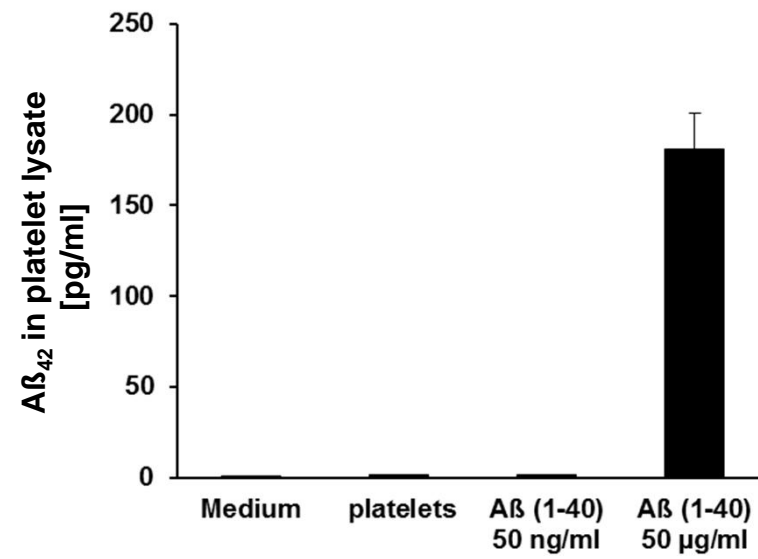

**Figure S1**

Supplement: Figure S1 — Accumulation of A ß (1–42) measured by a sandwich ELISA assay. Aß (1–42) levels measured in different control experiments: Medium alone, supernatant of resting platelets and medium supplemented with 50 ng/ml Aß (1–40) and 50 µg/ml Aß (1–40), respectively. Bar graphs depict mean values ±SEM (n = 4–8) of Aß1–42 levels as indicated. (PDF) [file pone.0090523.s001.pdf]

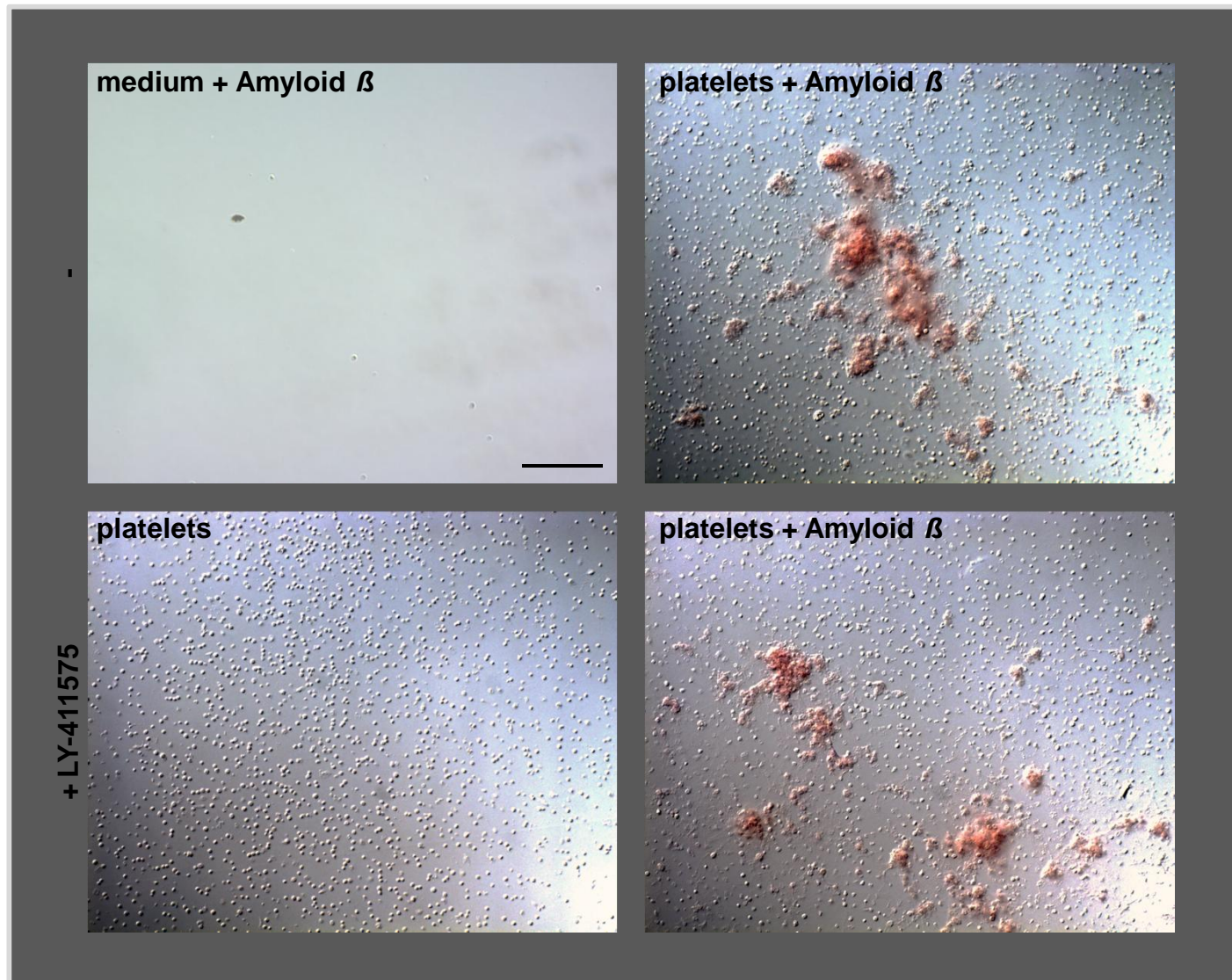

**Figure S2**

Supplement: Figure S2 — Formation of A ß deposits in murine platelet cell culture. Congo red-positive platelets and Aß deposits in platelet cell culture of C57BL/6J mice after stimulation with 50 µg/ml Aß in the presence and absence of the APP inhibitor LY-411575 after 10 days in culture. (right panel). Approaches with medium and Aß or platelets alone served as controls to confirm that Aß does not pre-aggregate to produce congo red-positive Aß deposits spontaneously. Scale bar 20 µm. (PDF) [file pone.0090523.s002.pdf]

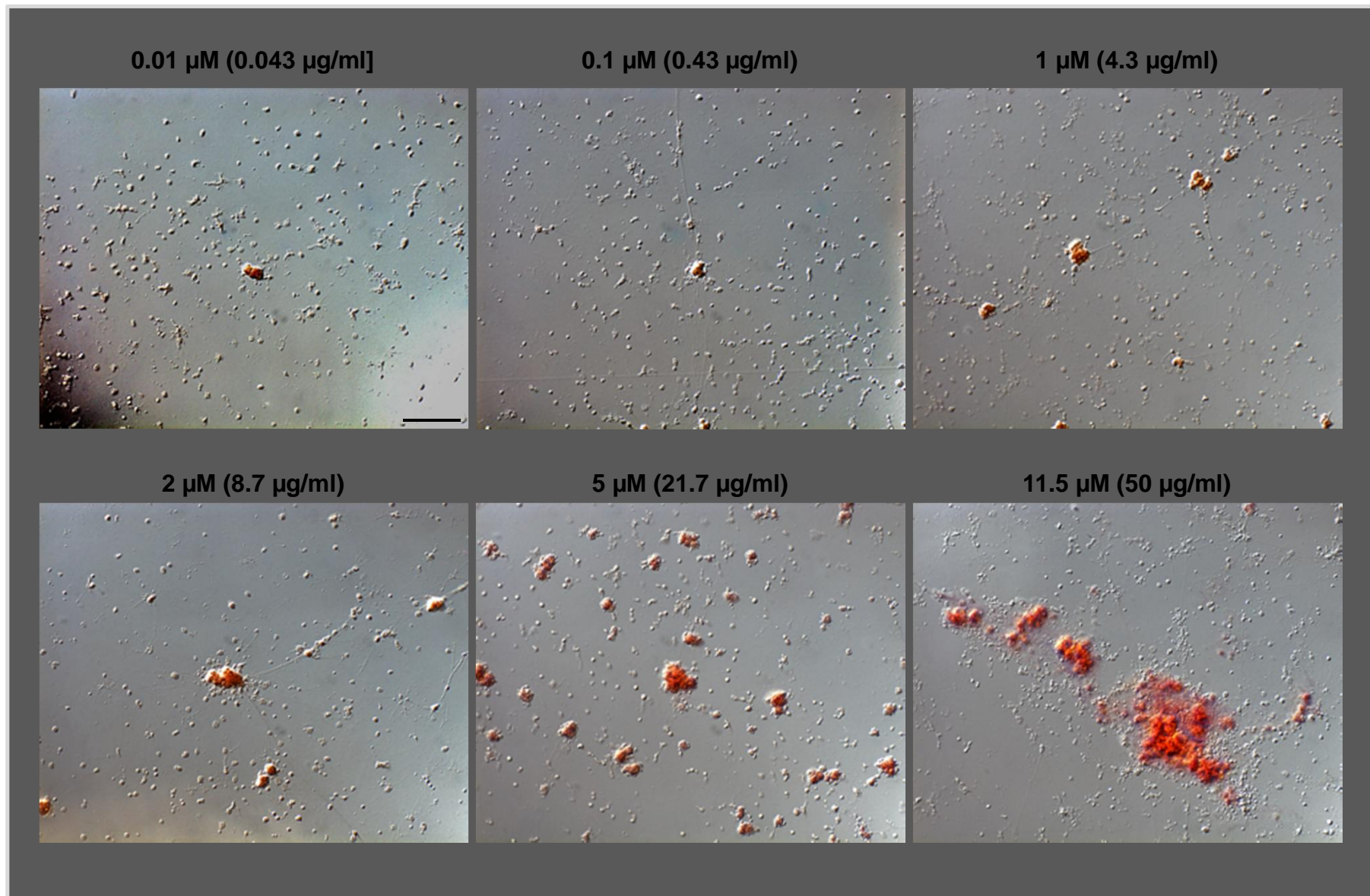

**Figure S3**

Supplement: Figure S3 — Platelet-mediated modulation of soluble A ß into congo red-positive fibrils is concentration-dependent. Congo red-positive platelets and Aß deposits in platelet cell culture after stimulation with indicated concentrations of soluble Aß after 10 days. Note the increase in extracellular Aß deposits with Aß concentrations >1 µM. Scale bar 20 µm. (PDF) [file pone.0090523.s003.pdf]
